# Tracing the wider impacts of biomedical research: a literature search to develop a novel citation categorisation technique

**DOI:** 10.1007/s11192-012-0642-8

**Published:** 2012-02-01

**Authors:** Teresa H. Jones, Claire Donovan, Steve Hanney

**Affiliations:** Health Economics Research Group, Brunel University, Uxbridge, Middlesex, UB8 3PH UK

**Keywords:** Research assessment, Citation categorisation, Methodology, Wider impacts of research, Citation generations

## Abstract

There is an increasing need both to understand the translation of biomedical research into improved healthcare and to assess the range of wider impacts from health research such as improved health policies, health practices and healthcare. Conducting such assessments is complex and new methods are being sought. Our new approach involves several steps. First, we developed a qualitative citation analysis technique to apply to biomedical research in order to assess the contribution that individual papers made to further research. Second, using this method, we then proposed to trace the citations to the original research through a series of generations of citing papers. Third, we aimed eventually to assess the wider impacts of the various generations. This article describes our comprehensive literature search to inform the new technique. We searched various databases, specific bibliometrics journals and the bibliographies of key papers. After excluding irrelevant papers we reviewed those remaining for either general or specific details that could inform development of our new technique. Various characteristics of citations were identified that had been found to predict their importance to the citing paper including the citation’s location; number of citation occasions and whether the author(s) of the cited paper were named within the citing paper. We combined these objective characteristics with subjective approaches also identified from the literature search to develop a citation categorisation technique that would allow us to achieve the first of the steps above, i.e., being able routinely to assess the contribution that individual papers make to further research.

## Introduction

Funders of health research increasingly recognise the need both to understand the translation of biomedical research into improved healthcare and to assess the extent to which these wider impacts or benefits to society are achieved (Buxton and Hanney 1996; Cooksey 2006). In the UK, the Medical Research Council, Wellcome Trust and Academy of Medical Sciences together created the UK Evaluation Forum who, in 2006, described the evaluation of medical research benefits as crucial to research stakeholders (UK Evaluation Forum 2006). Various research funders have already commissioned studies to show the wider impact that is made by the research that they have funded. These include, in the UK, public sector bodies such as the National Health Service (NHS) R&D Programme (Buxton and Hanney 1996) and medical research charities such as the Arthritis Research Campaign (arc) (Wooding et al. 2005). In terms of the techniques adopted, considerable progress has been made using case studies to assess the wider impact of biomedical research (Hanney et al. 2007; Wooding et al. 2005). These can use a variety of techniques, including bibliometric analysis, but a major element involves qualitative interviewing and, therefore, such studies are resource intensive. Furthermore, the UK Evaluation Forum recommended that further work be undertaken to develop methods to assess the benefits or payback from health research (UK Evaluation Forum 2006).

There are still debates about the best approach to use to assess the academic quality of research. There is increasing discussion about how far citation analysis can be used but traditionally, most citation analysis that is used in research evaluation relies on simple quantitative techniques which can be more mechanised and are not resource-intensive. How far such simple citation counts provide adequate measures of research quality has long been debated (Cave et al. 1988; Moed 2005; Research Evaluation and Policy Project (REPP) 2005). It is widely agreed, however, that such counts do not provide adequate assessments of wider benefits from health research (Allen et al. 2009; Jones et al. 2004). As Lewison (2004) observes, ‘both paper and patent citation counts may be inappropriate as measures of the practical effects of biomedical research.’

Some progress has, however, been made in assessing wider impacts by using novel citation approaches. Some studies start with specific documents and work back by analysing the papers cited on them. These documents have tended to be clinical guidelines (Grant et al. 2000) but such analysis could be extended to other policy documents or text books that could be part of a wider impact achieved by the health research (Lewison 2004). Nevertheless, there are limitations on how starting with specific documents and working back could, on its own, provide a way of assessing the wider impact of specific bodies of research.

Going the other way, working forwards from specific research and attempting to identify policy documents on which it is cited, can have a role in case studies (Buxton and Hanney 1996; Wooding et al. 2005) but on its own again it would be limited because it would not allow tracing of the impact made by one study on subsequent studies and through that route on to eventual wider impacts. Kostoff commented that ‘one largely unutilized role of citations is to serve as a “radioactive tracer” of research impacts…this is a very fruitful area for future citation research and analysis’ (Kostoff 1998). Also, Hu et al. (2011) claimed that ‘when studying a publication’s contribution to the evolution of its field or to science in general, taking only direct citations into account, tells only part of the story’.

Hanney et al. (2006) used a variety of both qualitative and quantitative techniques to identify a wide range of impacts from a body of health research. Their comparison of findings from the different methods used illustrated the inadequacies of using purely quantitative citation analysis for the task of identifying the wider impacts from research: some of the papers viewed by interviewees as having had an important impact on clinical practice did not receive many citations. In an attempt to move beyond purely quantitative citation analysis, one of the techniques developed in this study involved categorising citations received by a paper, to identify those where the cited paper was considered important (Hanney et al. 2005). In this approach Hanney et al. recorded details relating to each citation such as its location within the citing paper and the number of citing occasions, and also made an assessment of the reasons behind each citation occasion and the level of importance of the cited paper to the citing paper. This analysis suggested that just 9% of the citations received by a body of papers were considered to be of high importance to the papers citing them, and only 1% essential. Hanney et al. (2005) suggested that further work was needed.

In our current study we seek to build on the previous work and to develop a robust novel citation categorisation method and apply it to biomedical research in order to assess the contribution that individual papers make to further research by identifying those citing papers where the cited paper is of importance. Using this method we then propose to trace the important citations to the original research through a series of generations. Then we shall also attempt to identify the wider impacts made in any of the generations as a result of the papers being cited in clinical guidelines etc. This article describes the first part of the process, i.e., the steps taken to build on the previous study and develop our citation categorisation method. It describes our search of the bibliometrics’ literature and the way in which the search findings informed our subsequent development of a template that could be used to categorise citations.

### Method

For the development of our citation categorisation technique, we were particularly looking for ideas that would allow us quickly to examine all the citations given to a paper and identify those examples (probably few in number) where inclusion of the cited paper was of some importance to the citing paper. We also wanted to consider how we should manage self-citations. Furthermore, as we were not expecting to ask for authors’ opinions about why they cited particular papers as the basis of our method, it was particularly important to look for information that would help us to develop a method that could be applied not only quickly but also consistently by diverse assessors. We searched the literature for relevant information including empirical and other data to aid in the design of a novel approach to categorise citations based on their importance to the citing paper.

### Literature survey coverage

We conducted a comprehensive literature search both of citation databases and subject-specific databases for relevant articles (Tang and Safer, 2008). We included all years that the relevant databases were available up to October 2009, and all types of articles published in English (see Table 1). As we considered that the subject of our search was difficult to define precisely we attempted to increase the sensitivity of our survey by additionally conducting manual searches of four prominent journals (see Table 2) and by exploring the reference lists from eight key papers: two described above (Hanney et al. 2005; Kostoff 1998); a further five key papers describing both the positive and the negative motives behind citations (Case and Higgins 2000; Gilbert 1977; McCain and Turner 1989; Moravcsik and Murugesan 1975; Shadish et al. 1995) and Bornmann and Daniel (2008) who had more recently completed a major review of the reasons behind citations.

**Table 1:** Databases searched listed in the order of searching, details of the searches conducted and the dates covered by each search

Databases searched	Dates covered	Type of search	Extent of search	New papers identified after exact duplicates removed
WOS including SCI-expanded, SSCI, A&HCI, CPCI-S	1970–2009	Advanced	Title, abstract, author keywords, keywords plus	1,372
Scopus	1823–2009	Advanced	Title, abstract, keywords	640
Library, information science & technology abstracts (LISTA)	1965–2010	Boolean/phrase	Title, abstract, keywords	230
SpringerLink	All	Basic	Summary	710
Sigle	1980–2005	Search	Title, author, subject abstract, series, sponsor, identifier	31
Medline	1950–2009	Advanced	Title, abstract, subject heading	662
InfoSci journals	All	Basic	Abstract	13
Information science reference	All	Basic	Abstract	19

**Table 2:** Journals hand-searched and the dates covered in the search

Journals searched	Dates covered	Number of papers identified
Journal of documentation	1961–2009	679
Scientometrics	1978–2009	2,450
Journal of the American society of information science & technology	1972–2009	2,501
Social studies of science	1975–2009	519

### Search strategy

We sought to find publications that would provide potentially relevant information on the assessment or classification of the use of citations within a citing paper. The key terms used to develop an appropriate search strategy for each database were: analysis of citations; categorisation or assessment of the importance of citations; reasons for citing. Identified articles were transferred to an EndNote database in the order of searching with duplicates electronically removed on transfer. The number of new articles found in each database after the removal of duplicates was noted. In addition we collected all of the papers included in the journals in Table 2 and published between the dates listed and added them to the same EndNote database. Again duplicates were electronically removed on transfer.

### Review strategy

We chose to exclude papers that clearly were not relevant to our search by systematically reviewing each paper using the descriptions as listed in Table 3. Titles and abstracts were studied first and, where there was insufficient detail to make a decision, the full papers were obtained and the review process repeated to reach conclusions on relevance for each paper. Full papers for all those considered potentially useful were obtained and classified according to an iterative list of characteristics that was continuously developed throughout the review as informed by the research papers.

**Table 3:** Findings from our initial review of the papers identified in the searches described and reasons for exclusion from further study

Excluded papers: Total	8,765
Not a study of citations.	5,307
Only a quantitative assessment, e.g., a count of citations, mathematical manipulations such as Lotka’s law, Bradford, Hirsch; an assessment of a specific body of literature, e.g., as found in a journal, library, institution, geographical area; considering collaboration.	2,895
Only an assessment of the distribution of citations, e.g., co-citation, mapping, diffusion, data envelopment analysis, reference analysis.	496
Specifically describing: Ortega hypothesis; patents; comparisons of quantitative bibliometric techniques and peer-review; or paper not in English.	67
Papers for further study: Total	285
General background: theoretical papers concerning the meaning of citations without any relevant methodological detail but potentially interesting for our study.	179
Methodological: specific to the development of our method either in a general way or because they discuss particular issues such as self-citation or location of a citation.	106

## Findings

Details of the findings of our search strategy can be found in Tables 1 and 2. We identified 9,050 records after the automatic deletion of duplicates, 3,677 from the database searches, an additional 5,348 from journal hand-searches and a further 25 from the bibliography searches.

Findings from the first stage of the review procedure carried out on these 9,050 records are included in Table 3.

Through the survey we identified many research articles that were potentially of use to the development of our method although, apart from Hanney et al. (2005), no other research was identified that had attempted to explore Kostoff’s original suggestion (1998) of using citations to trace impacts across generations of citing papers.

### Meaning or use of citations

The considerable research that has previously been conducted on the use or meaning of citations within a citing paper covers various issues. For example, Gilbert (1977) discussed how citations could be considered as a method of persuasion for authors and Small (1978) regarded them as concept symbols. Numerous authors, e.g. (Case and Higgins 2000; Harwood 2009; Kostoff 1998; Oppenheim and Renn 1978; Peritz 1983; Shadish et al. 1995) have devised classification schemes for the use or meaning of citations. Moravcsik and Murugesan (1975) proposed a series of dichotomous questions for the classification procedure rather than an unrelated list of possible answers or an open question inviting authors to list their own reasons. The use or meaning of self-citations has also been widely discussed, e.g. (Garfield 1996; Snyder and Bonzi 1989; Tang and Safer 2008). In 2008, Bornmann and Daniel reviewed citation behaviour of scientists over a period of 15 years and concluded that whereas findings from the included studies varied widely in their design and that their results were ‘scarcely reliable’, there was some basis for the use of citations (Bornmann and Daniel 2008).

### The importance of citations

The importance of a citation to the citing paper has also been discussed, e.g. Safer and Tang (2009) used a scale of 1–7 for authors to indicate importance and found that only 9% of references contained a quote or discussed at least one point thoroughly. They also found that a further 11% mentioned the cited work in the text but in a limited way. Prabha (1983) also found the majority of citations to be of little importance as he identified that less than a third of cited papers were considered to be essential raw material to the citing papers. Researchers have widely investigated possible ways of characterising citations to indicate their influence, e.g. the location of a citation within a paper (Cano 1989; McCain and Turner 1989; Paul 2000; Peritz 1983; Safer and Tang 2009; Sombatsompop et al. 2006; Tang and Safer 2008) naming the first author (Paul 2000), the length of a citation (Tang and Safer 2008) or the number of occasions when a citation may be included in a citing paper (McCain and Turner 1989; Peritz 1983; Safer and Tang 2009; Sombatsompop et al. 2006; Tang and Safer 2008). Sombatsompop et al. (2006) combined the location of a citation within the paper with its significance on a four level scale within that section of the paper. Their findings indicated that citations in the Results and Discussion sections were comparatively more important than those in other sections. In some cases researchers asked authors to attach a level of significance to any reason that an author may have for including a citation (Case and Higgins 2000; Safer and Tang 2009; Shadish et al. 1995; Tang and Safer 2008).

### The citation assessment process

We identified research that had considered the practicalities involved in the assessment procedure, for example Peritz considered whether the use of the text surrounding the citation was adequate for assessment (Peritz 1983). The type of assessors carrying out the analysis of the citations has also been considered by many. Some literature considered the author’s views of why a citation has been included (Cano 1989; Harwood 2009; Prabha 1983; Tang and Safer 2008; White and Wang 1997). Hanney et al. (2005) had employed a number of assessors who were not the authors of the citing article, and the researchers examined the level of agreement between the assessors. In a slightly different but related context Moriarty et al. who were examining the sources of citations in cancer news articles, had considered the benefits of training assessors in preparation for a citation categorisation procedure (Moriarty et al. 2009).

## Discussion

Our literature search identified much discussion around the meaning and use of citations and also different considerations and methods for evaluating the importance within a citing paper of a particular citation. The literature search, therefore, could help us address a number of questions that we were facing in developing a citation analysis technique to apply to biomedical research in order to assess the contribution that individual papers made to further research. The issues we addressed included: how to filter out the large number of citations which are not really important to the cited paper; how to treat self citations; how to develop a template that could be applied quickly and consistently; how far objective characteristics of citations could be used to identify the citations that were important to the citing paper; how far we can identify appropriate subjective elements on which a qualitative assessment of importance will have to be made?

As we are proposing to trace the citations through up to six generations, there are potentially major questions about the feasibility of the overall project because the increase in the number of papers in the second and subsequent generations could be enormous and finding a way of managing it effectively could be a complex step in the assessment procedure. However, both our previous study and work identified in the literature search found that the numbers of citing papers for which the cited paper was highly important was only a small proportion of the total number of citations (Hanney et al. 2005; Prabha 1983). This is significant for our overall study but does mean that the identification of the citing papers for which the cited paper was highly important is a crucial practical step and, further, that this could require a multi-step assessment process.

We also examined discussions in the literature concerning the reasons behind self-citations, e.g. (Safer and Tang 2009; Snyder and Bonzi 1989; Tang and Safer 2008) and whether they should or should not be included in a citation assessment and, if included, whether they should be handled differently to non-self-citations (Safer and Tang 2009; Tang and Safer 2008). For quantitative citation analysis there is much concern about the potential for distortion of the outcome of the analysis should self-citations be considered in the same way as non-self-citations. However, some studies have concluded that when considering the wider impacts of research, authors often consider self-citations to be more important and informative to the research than non-self-citations, e.g. (Safer and Tang 2009; Snyder and Bonzi 1989; Tang and Safer 2008). Therefore, we concluded that at this stage self-citations should not be excluded from our study but data should be collected so that we could then more fully examine their role in tracing the wider impacts.

Concerns about the time required to conduct qualitative assessments, and the need for consistency were addressed in several of the identified papers. Peritz (1983) devised a categorisation method for the use of citations in empirical papers in the social sciences, specifically excluding historical studies, and considered that as little subjective judgement as possible should be included in the assessment process. In 2004, White considered that no citation classification scheme would be widely accepted unless its operation could be automated (White 2004). Therefore, we considered that objective data contained within a citing paper (e.g., location of citation, number of citation occasions) requires little evaluative judgement and so could be relatively simple and quick to collect accurately and also opened up the opportunity for possible future part-automation of the assessment process.

We identified various papers that analysed how far objective aspects of a research paper have been shown to relate to expert or author opinion of importance. Characteristics repeatedly found to be associated with expert or author opinion of the level of importance of a citation to the cited paper include the location of a citation and its frequency within the citing paper (Cano 1989; Peritz 1983; Safer and Tang 2009; Sombatsompop et al. 2006; Tang and Safer 2008). Characteristics that have been found to have some level of prediction of importance include naming of the first author (Paul 2000) and length of the citation (Tang and Safer 2008). However, these characteristics were found not to have the same predictive level of importance for self-citations (Safer and Tang 2009; Tang and Safer 2008). Of these characteristics, we considered that data on the location and frequency of citation as well as whether the author(s) were named could be straightforward to collect and therefore should allow good inter-rater reliability as well as speedy completion. The length of citation could have a subjective element involved but it also could potentially be incorporated. We chose to collect data relating to these characteristics to inform the design of a trial template and subsequently to evaluate their usefulness as part of our subsequent assessment procedure.

In considering the qualitative aspect of the assessment procedure we intended to use the method devised by Hanney et al. (2005) as the starting point. This approach had used a range of five possible reasons for the use of a citation and a 4-point scale of importance. Findings from the use of this method could be combined with studies identified in the literature, for example, the works of Case and Higgins’ (2000) and Shadish et al. (1995), to modify the characterisation scheme so as to allow the assessors to identify more easily the citing papers where the cited paper was of some importance rather than provide a full characterisation of the citation. We were not considering asking for authors’ opinions of the citations that they had used and the evidence provided by Haslam et al. (2008) who had employed graduate and post-graduate students to carry out some of the data collection on article organisation and had achieved good inter-rater reliability and Peritz’s (1983) conclusions that no more than a general acquaintance with the subject of the paper should be required from the assessors, we considered employing a group of post-graduates who were appropriately skilled. In light of Moriarty et al.’s findings (2009) we would provide familiarisation sessions and training for the post-graduates before the assessment procedure began. Inter-rater reliability would be measured using the Kappa coefficient and comparison of the assessors’ views via the assessment procedure would be compared with expert opinion and also with other methods available, generally of a more quantitative and/or automated nature.

Overall, therefore, the literature review helped us identify the objective and subjective elements to include in our categorisation of citations.

## Conclusions

Hanney et al. (2005) started developing an approach to put into practice an idea from Kostoff (1998) about using citations to trace the impacts of research. The literature reported here found no other attempts to apply a citation categorisation method to biomedical (or other) research in order to assess the wider impacts of research across many generations of citations. The survey, however, has revealed both objective and subjective techniques that could assist the development of a simple and informative assessment method to categorise citations.
